# Posttraumatic stress and growth in adolescent childhood cancer survivors: Links to quality of life

**DOI:** 10.3389/fpsyg.2022.992310

**Published:** 2022-09-09

**Authors:** Veronika Koutná, Marek Blatný, Martin Jelínek

**Affiliations:** ^1^Institute of Psychology, Czech Academy of Sciences, Brno, Czechia; ^2^Department of Psychology, Faculty of Arts, Masaryk University, Brno, Czechia

**Keywords:** posttraumatic stress, posttraumatic growth, benefit finding, quality of life, childhood cancer surivors

## Abstract

Pediatric cancer can be considered an event potentially leading to posttraumatic stress symptoms (PTSS) as well as posttraumatic growth (PTG). While clinically significant levels of PTSS are rare in childhood cancer survivors, PTG is common in this population. However, the relationship of PTG to overall adaptation and quality of life (QOL) in pediatric cancer patients is not clear. Therefore, our study aims to analyse the relationships of PTSS and PTG with QOL in childhood cancer survivors. In this study, 172 childhood cancer survivors completed measures of quality of life (*Minneapolis-Manchester Quality of Life Scale*; child and adolescent version), posttraumatic stress (*UCLA PTSD Reaction Index for DMS-IV*) and posttraumatic growth (*Benefit Finding Scale for Children*). Correlation analyses were carried out separately for the child (up to 13 years, *N* = 47) and adolescent (more than 13 years, *N* = 125) groups and each QOL dimension. In the adolescent group, the relationship of PTSS and PTG with QOL was further verified by regression analyses while controlling for age, gender, and time off treatment. In children, negative relationships between PTSS and QOL were found, but the relationships between QOL and PTG were not significant. In adolescents, significant relationships were found for all dimensions of QOL and PTSS and also for several dimensions of QOL and PTG. The relationships between PTSS and QOL dimensions were negative in both groups, and the relationships between PTG and QOL in the adolescent group were weakly positive. In adolescents, regression analyses controlling for age, gender and time off treatment were performed and confirmed a negative relationship of PTSS with all QOL dimensions except for social functioning. For PTG, regression analyses revealed a significant positive relationship with QOL dimensions of social functioning, outlook on life and intimate relations. While the relationship between PTSS and QOL is negative for almost all QOL dimensions in children and adolescents, the nature of the relationship between PTG and QOL appears to be more complex and changing over time. PTG in children may reflect different processes with different outcomes than PTG in adolescents.

## Introduction

Although childhood cancer is relatively rare and medical advances in recent years have led to an increase in the success rate of treatment, it is the second most common cause of death in childhood (following accidents; Ward et al., 2014). It is a life-threatening condition affecting the life of the whole family even after successful completion of treatment due to the risk of cancer recurrence and late effects. Therefore, long-term assessment of psychosocial adaptation of childhood cancer survivors has become one of the Standard of Care in Pediatric Oncology (Lown et al., 2015). As a part of this assessment, researchers frequently investigated the health-related quality of life (QOL) of survivors and the influence of various physical and psychosocial late effects of treatment. These studies showed that, in general, childhood cancer survivors do not usually report higher levels of anxiety, depression, emotional and behavioral difficulties or poor wellbeing (Yallop et al., 2013). Most of them do not reach clinically significant levels of posttraumatic stress (PTSS; Howard Sharp et al., 2015) and their QOL can be described as comparable with peers/healthy controls (Patenaude and Kupst, 2005; Harila et al., 2010; Blatný et al., 2011). Risk factors for PTSD and poor adaptation include female gender, problematic family background and diagnosis of a central nervous system tumour (Bruce, 2006). However, recent research has even found that a large proportion of childhood cancer survivors report positive consequences of their illness such as greater family closeness, reorganization of values or priorities and psychological maturity (e.g., Meyerson et al., 2011).

The positive consequences are usually described as posttraumatic growth (PTG) and occur in the domains of personal strength, relating to others, appreciation of life, new possibilities and spirituality (Calhoun and Tedeschi, 2006). Several studies (Meyerson et al., 2011; Turner et al., 2018; Berkman et al., 2020) analysed PTG correlates among demographic or psychosocial variables. However, it is not yet clear how the perception of positive change affects adaptation to trauma. While PTSS in childhood cancer survivors has been connected with higher levels of distress and poorer QOL (e.g., Meeske et al., 2001), the relationship of PTG with the overall adaptation seems to be more complicated.

Although intuitively it may seem obvious that PTG should have a positive relationship with overall adaptation, research in this area has produced conflicting results. In childhood cancer survivors, considerable attention has been devoted to the study of the PTG-PTSS relationship. Most of these studies operate with 4 possible forms of the relationship: positive, negative, curvilinear and PTG-PTSS as mutually independent (Casellas-Grau et al., 2017). Although some authors lean more toward the option of independence of PTG and PTSS (Klosky et al., 2014; Koutná et al., 2021), a recent systematic review found a positive relationship between PTG and PTSS (Marzilliano et al., 2019) in childhood cancer patients and survivors.

Some studies have also focused on the relationship between PTG and health-related QOL with similarly contradictory results and with different results for different dimensions of QOL. For example, a prospective follow-up study of adolescent and young adult cancer survivors (Husson et al., 2017) found a positive predictive effect of PTG on the mental dimension of QOL but no relationship between PTG and the physical dimension of QOL. This study also tested the interaction of PTG and PTSS in predicting QOL and found no interaction effect, which further supports the perspective of PTG and PTSS as two independent constructs. However, another study on pediatric cancer survivors (Kwak et al., 2021) and meta-analysis (Helgeson et al., 2006) did not find a relationship between PTG and QOL. A recent systematic review of adult cancer survivors (Liu et al., 2020) found a positive relationship between PTG and QOL.

The conflicting results in the studies of the PTG-QOL relationship may be related to the varying role of PTG with the growing amount of time since the trauma. PTG shortly after diagnosis was connected with poorer mental health and unrelated to physical health or depressive symptoms in adult survivors 3 months after diagnosis, but it predicted better physical health 3 months later (Tomich and Helgeson, 2012). Time since trauma was identified as a significant moderator of the relationship between PTG and adaptation (Helgeson et al., 2006). PTG early after diagnosis was connected with reduced negative symptoms such as depression and anxiety, whereas with a longer time interval from diagnosis, PTG was linked with better mental health (Sawyer et al., 2010). Therefore, some authors suggest that PTG early after trauma may reflect more of a coping style but with a longer time since trauma, it may reflect more substantial positive changes (Helgeson et al., 2006; Tomich and Helgeson, 2012).

In adult cancer survivors, the relationship between PTG and psychosocial adaptation appears to be further influenced by age. The positive relationship of PTG with mental health and well-being was found to be stronger for younger survivors, whereas in older survivors, there was a stronger negative connection between PTG and depression, anxiety, posttraumatic stress and distress (Sawyer et al., 2010).

Although there are several meta-analyses and review studies on the relationship between PTG and QOL, most of them focus on adult cancer patients. However, as noted above, the relationship of PTG and overall adaptation may depend on age. In addition to disease-related differences (e.g., incidence, type of cancer, treatment success and late effects), paediatric cancer patients may differ from adults in their ability to understand the disease and its consequences as well as strategies they use to deal with it. In the adult PTG model, the cognitive processing of a traumatic event called rumination plays a key role (Calhoun and Tedeschi, 2006). A prerequisite for this processing is a certain level of cognitive maturity. The minimum age at which children are capable of this processing is not clearly defined. Some authors consider the age of 11–12 years to be crucial for the development of cognitive abilities related to PTG (Turner et al., 2018).

This study aims to analyse the relationship of PTSS and PTG with individual dimensions of QOL in childhood cancer survivors. Based on the results presented above, we assume a negative PTSS-QOL relationship and a positive or neutral PTG-QOL relationship.

## Materials and methods

### Sample

The sample presented in this study is a part of the Quality of Life Longitudinal Study in Pediatric Oncology Patients (QOLOP) project. This project is focused on the longitudinal follow-up of the quality of life in long-term childhood cancer survivors and started in 2006. The project was approved by the Ethics Committee of the University Hospital Brno (02-300306/EK). Survivors were approached by the pediatric oncology clinic with an offer to participate in the study on the occasion of a regular check-up at the clinic (convenient sampling was performed). All survivors and/or their parents were thoroughly familiar with the aims, course and methods of the project and signed informed consent to participate in this study. All methods were administered to survivors in a paper-pencil form on the during the check-up. More details about the QOLOP project can be found in Blatný et al. (2011).

The sample used for the analyses included 172 childhood cancer survivors aged 11–25 (*m* = 16.76, SD = 3.64) at the time of assessment. This study is based on the cross-sectional data obtained in the second wave of data collection with a mean time off-treatment 8.55 years (SD = 2.72). A total of 217 survivors participated in the second wave of data collection. However, some of them were administered only a shortened version of the questionnaire set due to more severe cognitive late effects of treatment, and some did not provide complete data.

The sample in this study was almost balanced in terms of gender composition (53% males) and the majority of survivors were treated with leukemia or other solid tumors with no late effects. Only the minority was treated with CNS tumors (less than 15%) or suffered more serious late effects (less than 12%). For this study, the sample was divided into child and adolescent groups according to the version of the Minneapolis-Manchester Quality of Life Scale (MMQL) questionnaire used. The sample characteristics for these groups are presented in Table 1.

**TABLE 1:** Sample characteristics for child and adolescent groups.

		Child	Adolescent
		*N* = 47	*N* = 125
Gender *N* (%)	Male	21 (44.7)	70 (56.0)
	Female	26 (55.3)	55 (44.0)
Current age	*m* (SD)	12.33 (0.5)	18.42 (2.82)
	Range	11.5–13.7	14.0–25.3
Age at diagnosis	*m* (SD)	3.72 (2.01)	8.18 (3.93)
Time off-treatment (years)	*m* (SD)	7.17 (1.55)	9.07 (2.88)
Diagnosis *N* (%)	CNS	5 (10.6)	20 (16.0)
	Leukemia	27 (57.4)	41 (32.8)
	Other	15 (31.9)	64 (51.2)
Late effects *N* (%)	No	30 (63.8)	52 (41.6)
	Mild	13 (27.7)	42 (33.6)
	Moderate	1 (2.1)	19 (15.2)
	Severe	3 (6.4)	12 (9.6)
PTSS	*m* (SD)	1.44 (0.43)	1.69 (0.55)
PTG	*m* (SD)	3.25 (1.02)	3.59 (0.93)

CNS, central nervous system.

### Methods

Minneapolis-Manchester Quality of Life Scale (MMQL) was used for the assessment of QOL. MMQL is a disease-specific measure of QOL in cancer patients and two age-appropriate versions reflecting different needs and language abilities of different age groups were used in this study. MMQL-YF (Bhatia et al., 2004) is designed for survivors from 8 up to 12 years and includes 32 items divided into 4 subscales: outlook on life and family dynamics (e.g., looking forward to the future, Cronbach’s alpha = 0.744), physical symptoms (e.g., pain, Cronbach’s alpha = 0.685), physical functioning (e.g., have a lot of energy, Cronbach’s alpha = 0.720) and psychological functioning (e.g., feeling sad, Cronbach’s alpha = 0.667). The total Cronbach’s alpha for MMQL-YF = 0.612. MMQL-Adolescent form (Bhatia et al., 2002) is intended for survivors older than 13 years and includes 46 items divided into 7 subscales: outlook on life (e.g., happy with life in general, Cronbach’s alpha = 0.806), physical functioning (e.g., feeling strong and healthy, Cronbach’s alpha = 0.804), psychological functioning (e.g., worried about health, Cronbach’s alpha = 0.825), social functioning (e.g., have many close friends, Cronbach’s alpha = 0.824), cognitive functioning (e.g., difficulty in concentrating, Cronbach’s alpha = 0.782), body image (e.g., being happy about the way they look, Cronbach’s alpha = 0.824) and intimate relations (e.g., difficulty in making friend, Cronbach’s alpha = 0.768). The total Cronbach’s alpha for MMQL-Adolescent form = 0.903. Survivors report their QOL on a 4 or 5-point scale. The MMQL-Adult form was not available at the time of data collection, therefore the MMQL-Adolescent version was used without the upper age limit. The MMQL-YF was exceptionally (in 5 survivors) administered to 13-year-olds according to the recommendation of the clinic staff because its shorter and simpler form was more suitable for them.

The University of California at Los Angeles Posttraumatic Stress Disorder Index for DSM-IV (UCLA_PTSD) was used for the assessment of PTSS. The questionnaire measures the frequency of individual symptoms of posttraumatic stress as defined in the DSM-IV (avoidance, re-experiencing and increased arousal) in the past month (Pynoos et al., 1998; Steinberg et al., 2004). The DSM-IV version was used because it was the current version at the time data collection began. The reliability of UCLA_PTSD in this study was good (Cronbach’s alpha = 0.907). Survivors report the frequency of PTSS on a 5-point scale. In this study, the experience of illness was explicitly identified as the event to which each questionnaire item relates. For example, instead of item “When something reminds me of *what happened*, I get very upset, afraid or sad” we used item “When something reminds me of *my disease*, I get very upset, afraid or sad,” or instead of “I have dreams about *what happened* other bad dreams” we used “I have dreams about *my disease* or other bad dreams.”

Benefit Finding Scale for Children (BFSC) was used to assess PTG in childhood cancer survivors. BFSC is a frequently used measure in PTG research and consists of 10 items addressing positive change on a 5-point scale (Phipps et al., 2007; Michel et al., 2009). The reliability of BFSC in this study was good (Cronbach’s alpha = 0.908).

Ale methods were administered to the survivors in Czech language. The severity of late effects was evaluated according to Common Terminology Criteria for Adverse Events v3.0 so that the most serious of the occurring late effects was decisive to the resulting degree of severity. The evaluation was performed by a physician.

### Statistical analysis

To analyse the relationship between PTSS/PTG and QOL, correlation analyses were carried out separately for the children (MMQL-YF) and adolescent (MMQL-Adolescent form) groups and individual MMQL dimensions. In the adolescent group, the relationship of PTSS and PTG with QOL was further verified by regression analyses while controlling for age, gender, and time off treatment. The children group was not further analysed due to the small sample size. All analyses were performed using SPSS 27.

The sample was divided in two age groups based on the version of MMQL. Due to differences in the number of subscales and items, item wording as well as response scale between MMQL_YF and MMQL-Adolescent form, data obtained by this method cannot be merged and we treat them separately. In addition, the age limit of 12 years, which determines the use of these two versions of MMQL, represents a developmental milestone in terms of the development of PTG-related cognitive abilities (Turner et al., 2018).

## Results

The sample for this study includes 172 survivors divided into child (*N* = 42) and adolescent (*N* = 125) groups analysed separately due to different versions of MMQL. More details about these groups can be found in Table 1. In the comparison of the child and adolescent group, adolescent survivors reported higher levels of both PTSS (*t* = –3.20^**^) and PTG (*t* = –2.03*). Based on the 38 cut-off PTSS score (Steinberg et al., 2004), a clinically significant level of PTSS was reported by 10 (6.2%) survivors.

The results of the correlation analysis of PTSS and PTG with individual QOL dimensions are presented in Tables 2, 3. In both age groups, PTSS was connected to decreased QOL in all dimensions. On the other hand, significant relationships between PTG and QOL were found only in the adolescent group. Adolescents with higher levels of PTG report higher scores on the outlook of life (life satisfaction) and cognitive functioning dimensions of QOL.

**TABLE 2:** Correlation analyses for the child group (*N* = 47).

	PTG	Physical symptoms	Outlook on life	Physical functioning	Psychological functioning
PTSS	0.010	0.324*	−0.603**	−0.499**	−0.576**
PTG	–	0.045	–0.073	0.036	–0.108
Physical symptoms		–	–0.211	−0.384**	–0.288
Outlook on life			–	0.611**	0.491**
Physical functioning				–	0.373**

*p < 0.05; **p < 0.01.

**TABLE 3:** Correlation analyses for the adolescent group (*N* = 125).

	PTG	Outlook on life	Physical functioning	Psychological functioning	Cognitive functioning	Body image	Social functioning	Intimate relations
PTSS	−0.117	−0.467**	−0.433**	−0.668**	−0.568**	−0.471**	−0.179*	−0.272**
PTG	−	0.205*	–0.022	0.020	0.190*	0.063	0.161	0.159
Outlook on life		−	0.460**	0.393**	0.378**	0.480**	0.454**	0.374**
Physical functioning			−	0.519**	0.368**	0.406**	0.414**	0.408**
Psychological functioning				−	0.507**	0.422**	0.279**	0.370**
Cognitive functioning					−	0.424**	0.242**	0.271**
Body image						−	0.323**	0.396**
Social functioning							−	0.568**

*p < 0.05; **p < 0.01.

Table 4 presents the result of regression analyses controlling for age, gender a time off-treatment when analysing PTSS/PTG-QOL relationship in the adolescent group. Models for all QOL dimensions were significant explaining 10–47% of the variance (the lowest level of explained variance was found for the domain of social functioning, and the highest level of explained variance was found for the domain of psychological functioning). The analyses confirmed the negative relationship of PTSS with all dimensions of QOL except for social functioning. The strongest PTSS-QOL relationship was found for the psychological functioning domain (β = –0.682), the weakest for the intimate relations domain (β = –0.237). In the case of PTG, the relationships with the outlook on life (life satisfaction), social functioning and intimate relations yielded significant results – survivors with a higher level of PTG report greater life satisfaction, better social functioning and more confidence in intimate relations. In the case of outlook on life and intimate relations, both PTSS and PTG significantly contributed to the final model. Age was a significant individual predictor of outlook on life and social functioning domains of QOL (younger survivors report higher QOL in these domains). Gender was a significant individual predictor for the domain of intimate relations (boys reporter higher level of satisfaction in intimate relations). Time since treatment completion was not a significant predictor for any of the QOL domains.

**TABLE 4:** Regression analyses controlling for age, gender a time off-treatment in the adolescent group (*N* = 125).

	Physical functioning	Psychological functioning	Outlook on life	Cognitive functioning	Social functioning	Body image	Intimate relations
	β	β	β	β	β	β	β
Age	–0.087	–0.054	−0.166*	–0.022	−0.212*	–0.036	–0.057
Gender	–0.107	–0.060	0.070	0.069	–0.105	0.065	−0.197*
Time off treatment	0.081	–0.130	–0.027	–0.003	0.054	–0.044	0.023
PTSS	−0.427**	−0.682**	−0.461**	−0.561**	–0.151	−0.480**	−0.237**
PTG	–0.018	–0.038	0.178*	0.113	0.230*	–0.002	0.195*
	*R^2^* = 0.214**	*R^2^* = 0.474**	*R^2^* = 0.275**	*R^2^* = 0.344**	*R^2^* = 0.100*	*R^2^* = 0.229**	*R^2^* = 0.128*

*p < 0.05; **p < 0.01.

For gender: 1 = female, 0 = male.

## Discussion

This study aimed to clarify the relationship between PTSS/PTG and QOL in long-term childhood cancer survivors with taking into account individual dimensions of QOL and basic demographic factors which are known to be related to PTSS, PTG and QOL: age, gender and time off-treatment.

Correlation analysis revealed negative relationships for PTSS and all domains of QOL for both age groups. The connection of elevated levels of PTSS with lower QOL is in line with previous research (Meeske et al., 2001). The strength of most of these correlations ranged from medium to high. Only the correlations with social functioning and intimate relations were rather weak. For PTG, a significant positive relationship was found only in the older age group and only for two domains of QOL – outlook on life and cognitive functioning. Although these relationships were less tight than in the case of PTSS, we can assume that the PTG-QOL relationship evolves with age. While no association has been found between QOL and PTG in children, the relationship between PTG and QOL begins to emerge in adolescents. Therefore, we believe that PTG in children may be of a different nature than PTG in adolescents/young adults. Finding meaning in a traumatic event requires a certain level of cognitive maturity that cannot be expected from children. Some authors date the development of these abilities to early adolescence in the period of 11–12 years (Turner et al., 2018). This is consistent with the results of the present study, in which adolescents reported higher PTG scores compared to children. As cognitive abilities develop, adolescents may be better able to assess the impact of the disease on their lives (Blatný et al., 2013), and this more accurate assessment can then be reflected in their quality of life and overall adaptation.

When analysing the PTSS-QOL relationship using regression analysis controlling for age, gender and time off-treatment in the adolescent group, the significant relationship of PTSS with QOL was confirmed for all dimensions except for social functioning. In contrast, social functioning was one of the few dimensions with a significant relationship with PTG. This may reflect the fact that interpersonal relationships belong to the dimensions in which PTG typically occur (Calhoun and Tedeschi, 2006) – improvements in relating to others as a part of PTG may reflect in better social functioning domain of QOL. However, social functioning was also the domain with the lowest level of explained variance. More research is needed to analyse the importance of other factors contributing to the social aspects of QOL.

PTG was also associated with a better outlook on life (life satisfaction) and intimate relations. The intimate relations subscale is very close in content to the social functioning subscale and its association with PTG could also be explained by the fact that interpersonal relations represent one of the fundamental dimensions of PTG. However, the association of PTG with these QOL dimensions may also be explained by the fact that patients with greater satisfaction in social relationships and higher levels of social support may be more likely to report positive changes following trauma. Social support has been connected with PTG in several studies (e.g., Prati and Pietrantoni, 2009).

We can think in a similar way about the connection between PTG and outlook on life. In addition to improved interpersonal relationships, PTG often results in a change in the philosophy of life and reorganization of priorities (Calhoun and Tedeschi, 2006). These changes are usually described as changes in what is perceived as important in life, greater respect for life, awareness of the importance and the joy of ordinary little things etc. This new perspective can then translate into greater life satisfaction. Perhaps these findings could be perceived as overlaps of PTG and QOL rather than their associations. Positive changes in dimensions such as interpersonal relationships or changed philosophy of life may be reflected in subjective evaluations of corresponding QOL domains and life satisfaction.

Our study did not find an association between PTG and physical, psychological and cognitive functioning or body image. Unlike social functioning, intimate relations and outlook on life, these QOL dimensions are not as directly represented in the areas typical for PTG. Instead, these dimensions of QOL were more closely related to PTSS. After a closer examination of individual items in the MMQL questionnaire, a part of these connections is quite easily explainable. The items of the psychological functioning subscale are narrowly focused on the presence of negative emotions. Negative cognitions and moods form a separate symptom cluster of PTSS in the DSM-5 (American Psychiatric Association, 2013). Intrusive thoughts as another PTSS symptom cluster together with negative moods can interfere with the cognitive functioning of survivors and their ability to concentrate. Although the association between physical functioning and body image with PTSS cannot be explained so straightforwardly, the lack of energy and uncertainty about one’s health and appearance fit in with negative beliefs or expectations about oneself typical of posttraumatic stress (American Psychiatric Association, 2013). Moreover, the lack of association between PTG and the physical domain of QOL is in line with the literature (Husson et al., 2017). From this point of view, it seems logical that these subscales are more strongly related to PTSS than to PTG.

Taken together, our results show that the relationship between PTSS and QOL in childhood cancer survivors is more pronounced than the relationship between PTG and QOL. The relationship of PTG with QOL was found only in some dimensions of QOL and was rather weak, but was not negative for any dimension. Thus, PTG is not necessarily always associated with better overall QOL, but neither is it associated with reduced QOL. In addition, the nature of the PTG-QOL relationship is not universal, it may change with the age of survivors.

Our results also offer several implications for clinical practice. The finding that PTG is not necessarily reflected in better QOL implies that psychosocial support may be suitable even for survivors who perceive benefits in their experience. Even their QOL may be compromised in some dimensions. Perception of the positive consequences of a traumatic event does not diminish the negative impact of this event. PTG can co-exist with PTSS (Calhoun and Tedeschi, 2006) and this must be kept in mind when planning psychosocial care for childhood cancer survivors. Moreover, some authors (e.g., Zoellner and Maercker, 2006) think of PTG more in terms of a positive illusions with possible negative consequences, and our results suggest that while PTG may not always be beneficial, it is probably not harmful either.

This study has some limitations that must be kept in mind when interpreting the results. First of all, it is a cross-sectional study, which prevents us from drawing conclusions about causality. The relationships identified by our results must be considered in both directions. Survivors with higher levels of PTG report better QOL in several dimensions. Or the other way round, those with better QOL report more benefits from their experience. Longitudinal studies are needed to understand the causal link between PTG and QOL. The sample of this study includes only 47 survivors in the children group compared to 125 adolescents and our results regarding children must be considered preliminary. More research with higher sample size is needed to verify these findings. Furthermore, the majority of our sample reported low levels of PTSS, which is comparable with results reported in the reviews of PTSS in childhood cancer survivors (Taïeb et al., 2003; Bruce, 2006) and indicative of their good overall psychosocial adaptation (Patenaude and Kupst, 2005). However, our results need to be verified in the sample with a higher prevalence of clinically significant levels of PTSS in studies using a more up-to-date method for assessing PTSS. Future research should also focus on the specifics of PTG processes and outcomes in children and adolescents and their association with overall adaptation or quality of life as well as on the identification and development of key cognitive abilities in relation to PTG.

## Data availability statement

The datasets presented in this article are not readily available because participants of this study did not agree for their data to be shared publicly. Requests to access the datasets should be directed to VK, koutna@psu.cas.cz.

## Ethics statement

The studies involving human participants were reviewed and approved by Ethics Committee of the University Hospital Brno. Written informed consent to participate in this study was provided by the participants’ legal guardian/next of kin.

## Author contributions

VK and MB: conceptualization and writing – review and editing. MJ: data curation. VK and MJ: formal analysis. MB and MJ: methodology. MB: supervision. VK: writing – original draft preparation. All authors have read and agreed to the published version of the manuscript.
